# The circITSN1/eIF4A3/Itsn1 axis, a potential new molecular entry point for managing postoperative cognitive dysfunction

**DOI:** 10.1016/j.omtn.2025.102674

**Published:** 2025-08-29

**Authors:** Sedef Ersoy, Christian Bär

**Affiliations:** 1 Institute of Molecular and Translational Therapeutic Strategies, Hannover Medical School, Hannover, Germany; 2 Fraunhofer Institute for Toxicology and Experimental Medicine (ITEM), Hannover, Germany; 3 Fraunhofer Cluster of Excellence Immune-Mediated Diseases (CIMD), Hannover, Germany

## Introduction

Postoperative cognitive dysfunction (POCD) is the impairment of cognitive abilities in elderly patients after surgery. Among others, it encompasses neurophysiological changes and therefore impairment in the ability to learn, think, and memorize.^1^ While there are some patients with a risk of developing a form POCD that persists for more than a year, most cases show a reversible phenotype, with cognitive abilities being restored after several weeks or months.^2^ As there is no specific treatment, however, to speed up the healing process, prevention strategies are of great importance.^1^

In their recent study, Zhang et al. deepened the understanding of these mechanisms by identifying a novel regulatory pathway with circular RNAs (circRNAs), circITSN1, as one of the key players. The authors demonstrate that POCD is promoted by circITSN1 in aged mice after surgery by binding to eIF4A3 and stabilizing Itsn1 mRNA, which is derived from the same host gene. Ultimately, this process leads to the activation of the c-Jun N-terminal kinase (JNK) inflammatory pathway and thereby to the manifestation of POCD. This study does not only deepen the understanding of molecular mechanisms involved in the development of POCD but also provides a new therapeutic target for clinical applications.^3^

### Circular RNAs in neurodegeneration and POCD

In recent years, there has been increasing interest in the roles of circular RNAs (circRNAs) in the pathophysiology of the central nervous system.^4^ CircRNAs are noncoding RNAs that form covalently closed loop structures and therefore lack free 5′- and 3′-ends. They are derived from pre-mRNAs and are formed through a process called backsplicing. This is opposed to alternative splicing which generates linear mRNAs with variable exon composition. Being not as prone to exonucleases as their linear counterparts, they show higher stability due to the lack of free ends.^5^ They are highly expressed in neural tissue,^5^ and several studies imply a role in Alzheimer’s disease, Parkinson’s disease, and stroke.^4^ Their functions range from microRNA or protein sponging to interaction with RNA-binding proteins. Additionally, circRNAs can play a role in modulating transcription or translation or as biomarkers for certain diseases.^5^ Since circRNAs mainly derive from protein-coding genes, they are often remarkably well conserved between species, especially compared to other types of noncoding RNAs such as long non-coding RNAs, making them increasingly attractive from a translational perspective for clinical applications.^5^ In the context of POCD, circRNAs could function as important regulatory factors: Especially in elderly patients, surgical trauma can cause systemic and local inflammatory processes. These in turn, can then lead to leakiness of the blood-brain barrier and to impairment of hippocampal function. It is implied that these processes are induced and mediated by damage-associated molecular patterns (DAMPs) and the subsequent inflammatory response in the central nervous system.^6^ There is emerging evidence that circRNAs could act as DAMPs or regulatory factors in these inflammatory responses.^7^

The work by Zhang et al. addresses the important gap of functionally validating circRNAs as causal factors in POCD. This advance provides a mechanistic framework for understanding how circRNAs contribute to neuroinflammation and cognitive decline.^3^

### Key findings and mechanistic insights

In their study, Zhang et al. identify hsa_circRNA_061570, which was significantly upregulated in the plasma of elderly patients, who developed POCD after anesthesia and surgery. The authors worked with a murine POCD model of aged mice after unilateral nephrectomy, where the murine homolog, circITSN1, is similarly increased. To validate the suitability of their POCD mouse model, Zhang and colleagues performed behavioral tests such as Y maze or fear conditioning, which confirmed hippocampus-dependent memory deficits in surgically treated animals.

After confirming the upregulation of circITSN1 in the hippocampus and in serum of the POCD mice, the authors performed adeno-associated virus-short hairpin RNA-mediated hippocampal knockdown of circITSN1 in their mouse model, which led to improved cognitive function. This was accompanied by improvements in dendritic spine injury; increased levels of postsynaptic density protein-95 and synaptophysin; and reduced hippocampal expression of pro-inflammatory cytokines like interleukin (IL)-1β, IL-6, or tumor necrosis factor alpha in the POCD mice.

Mechanistically, Zhang and colleagues show that circITSN1 binds directly to eukaryotic initiation factor 4A-III (eIF4A3), a translation initiation factor and RNA-binding protein involved in exon junction complex formation and mRNA transportation.^8^ The authors proved that this interaction enhances the stability of Itsn1 mRNA, which encodes Intersectin-1, a scaffolding protein with known functions in neuronal maintenance.^9^ In turn, upregulation of Itsn1 activated the JNK signaling cascade, a well-known mediator of cellular stress responses and neuroinflammation.^10^

**Figure 1:**
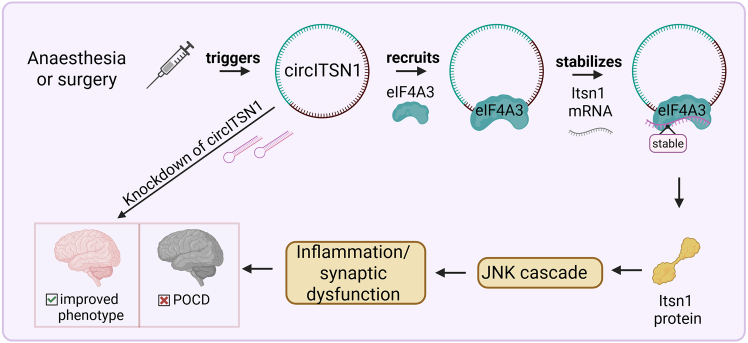
Causality of manifestation of postoperative cognitive dysfunction Murine CircITSN1 becomes upregulated upon anesthesia or surgery and recruits eIF4A3. This complex stabilizes Itsn1 mRNA and causes translation of Itsn1 protein, which in turn results in activation of the JNK cascade and thereby in inflammation and synaptic dysfunction, finally resulting in the manifestation of POCD.

When knocking down Itsn1 itself, the linearity of the whole process could be confirmed and the beneficial effects of knocking down circITSN1 were proved. Taken together, the authors could show that circITSN1 binds directly to eIF4A3 and thereby leads to stabilization of Itsn1 mRNA. This in turn induces the activation of the JNK signaling pathway, eventually leading to the manifestation of POCD (Figure 1).

### Broader significance and implications

The study offers several important contributions to deepen the understanding of circRNAs in the context of POCD. While most circRNAs are associated with different diseases, few have been functionally validated as causal. In this study, circITSN1 is established as a key modulator in POCD pathology. Further, the circITSN1/eIF4A3/Itsn1 axis represents a previously unrecognized mechanism for the regulation of neuronal inflammation and synaptic dysfunction on a post-transcriptional level.

Another important implication is the therapeutic and diagnostic potential of circITSN1. As this murine circRNA is elevated in both tissue derived from the hippocampus and serum samples, circITSN1 shows a potential use as a biomarker for POCD. Indeed, the authors showed that the human homolog hsa_circRNA_061570 was also upregulated in human serum samples derived from POCD patients post-surgery.

Due to its circular structure and therefore its stability, circITSN1 might additionally be feasible for clinical assays. Lastly, the study suggests the development of new therapeutic strategies to knock down circITSN1 or its downstream effectors and thereby decrease the burden of POCD.

### Future directions

While this study represents an important mechanistic model, some questions remain to be answered. The findings in this work are derived from cultured cells or murine models, meaning that the relevance of circITSN1 in the human central nervous system warrants further validation. Furthermore, the question remains, how the temporal expression of circITSN1 changes after surgery, and how comparable this is between individuals. Lastly, the functional role of circITSN1 may extend beyond eIF4A3 and Itsn1 mRNA. Whether this circRNA acts as a microRNA sponge or plays other roles in different regulatory networks remains an open question. When looking into future perspectives, the question arises whether circITSN1 could indeed become a clinical tool for the treatment or even prevention of POCD. Its inherent stability due to its circular structure along with its involvement in cognitive decline suggests potential as both a biomarker and a therapeutic target. For these future directions to become possible, translational research in patient cohorts will be needed. Nonetheless, this work provides a strong experimental foundation for bridging circRNA biology to post-surgical brain health.

## Acknowledgments

This work was supported by the Deutsche Forschungsgemeinschaft DFG (SFB TRR267 grant no: 403584255). The figure in this commentary was created using BioRender. https://BioRender.com/98xnibv.

## Declaration of interests

C.B. filed patents claiming the use of noncoding RNAs as therapeutic target and/or as disease biomarker.
